# Maternal Cytomegalovirus Viral Load Correlates With the Risk of Congenital Infection and With Antiviral Therapy Response in Early Pregnancy

**DOI:** 10.1093/ofid/ofag291

**Published:** 2026-06-24

**Authors:** Charles Egloff, Christelle Vauloup Fellous, Nadhira Houhou-Fidouh, Alexandra Benachi, Laurent Mandelbrot, Alexandre J Vivanti, Olivier Picone

**Affiliations:** Assistance Publique-Hôpitaux de Paris APHP.Nord, Service de Gynécologie Obstétrique, Hôpital Louis Mourier, Colombes, France; Université Paris Cité, Paris, France; Groupe de Recherche sur les Infections Pendant la Grossesse (GRIG), Velizy, France; Groupe de Recherche sur les Infections Pendant la Grossesse (GRIG), Velizy, France; INSERM U1184, CEA, Center for Immunology of Viral, Auto-Immune, Hematological and Bacterial Diseases (IMVA-HB/IDMIT), Paris Saclay University, Fontenay-aux-Roses, France; Assistance Publique-Hôpitaux de Paris, Division of Virology, Laboratoire de Biologie Médicale de Référence “Virus et Périnatalité” “Paul Brousse” Hospital, Paris Saclay University Hospitals, Villejuif, France; Assistance Publique-Hôpitaux de Paris, Virology Department, Bichat Hospital, Université de Paris, Paris, France; Assistance Publique-Hôpitaux de Paris, Department of Obstetrics and Gynecology, DMU Santé des Femmes et des Nouveau-nés, Antoine Béclère Hospital, Paris Saclay University, Clamart, France; Assistance Publique-Hôpitaux de Paris APHP.Nord, Service de Gynécologie Obstétrique, Hôpital Louis Mourier, Colombes, France; Université Paris Cité, Paris, France; Groupe de Recherche sur les Infections Pendant la Grossesse (GRIG), Velizy, France; INSERM, IAME, Paris, France; Groupe de Recherche sur les Infections Pendant la Grossesse (GRIG), Velizy, France; Assistance Publique-Hôpitaux de Paris, Department of Obstetrics and Gynecology, DMU Santé des Femmes et des Nouveau-nés, Antoine Béclère Hospital, Paris Saclay University, Clamart, France; Assistance Publique-Hôpitaux de Paris APHP.Nord, Service de Gynécologie Obstétrique, Hôpital Louis Mourier, Colombes, France; Université Paris Cité, Paris, France; Groupe de Recherche sur les Infections Pendant la Grossesse (GRIG), Velizy, France; INSERM, IAME, Paris, France

**Keywords:** biomarkers, cytomegalovirus, DNAemia, pregnancy, valacyclovir

## Abstract

**Background:**

To evaluate the impact of maternal cytomegalovirus (CMV) viral load at the time of intervention by valacyclovir (VCV) in preventing maternal–fetal transmission following primary CMV infection in early pregnancy and to assess the effect of VCV on maternal viral load kinetics.

**Method:**

We conducted a retrospective, observational, dual-center study including pregnant women referred for suspected primary CMV infection during the periconceptional period or first trimester, based on serological screening performed. Primary infection was subsequently confirmed according to predefined criteria. Maternal viral load was measured by quantitative PCR on whole blood at the time of initial evaluation in the perinatal center, before treatment initiation. Patients were analyzed according to viral load status (negative, detectable, or quantifiable) and whether they received high-dose oral VCV (8 g/day). The primary outcome was congenital CMV infection, confirmed by neonatal urine PCR. Secondary outcomes included maternal viral load kinetics and their relationship to treatment.

**Results:**

A total of 133 patients were included (90 VCV, 43 untreated). At diagnosis (median 14 weeks of gestation), 31 (24%) had negative viral load, 48 (36%) detectable, and 54 (40%) quantifiable viral load. No congenital CMV infections occurred in patients with negative viral load, regardless of treatment. Among those with quantifiable viral load, VCV was associated with lower risk of transmission compared to untreated controls (21% vs 58%; *P* = .028). For detectable but not quantifiable viral load, transmission rates did not differ. Longitudinally, viral load declined faster in treated patients (slope −0.3059 vs −0.1334; *P* = .003).

**Conclusions:**

Maternal CMV viral load may serve as a useful prognostic indicator after primary infection in early pregnancy. Its presence could help identify a subgroup at increased risk of vertical transmission who might benefit from VCV. Conversely, negative viral load appears to be associated with a lower likelihood of fetal infection.

## Teaser

Maternal viral load predicts congenital cytomegalovirus transmission risk. Valacyclovir significantly reduces maternal-fetal transmission only when viral load is quantifiable. Negative maternal viral load indicates minimal transmission risk, supporting routine CMV PCR testing in early pregnancy to guide antiviral therapy decisions in prevention of maternal-fetal transmission.

Congenital cytomegalovirus (CMV) infection is common, occurring in approximately 0.5%–1% of live births in the United States and Europe, and is the most prevalent congenital viral infection and a leading cause of neurological disorders and non-genetic hearing loss [1].

Several studies have demonstrated that treatment with valacyclovir (VCV) reduces maternal–fetal transmission, particularly after periconceptional and first-trimester maternal primary infection (PI), a period associated with the highest risk of fetal injury [2–4]. It would appear that the earlier VCV is started after infection, the more effective it appears to be [2]. Additionally, few studies have investigated the prognostic factors influencing maternal–fetal transmission prior to the introduction of VCV.

We previously reported that the efficacy of VCV was primarily associated with the presence of maternal viral load [5]. However, our study had several limitations: it included cases up to the second trimester of pregnancy, and the analysis did not account for the interval between PI and the onset of viral load or its quantification.

The objective of this study was to assess the impact of maternal CMV viral load at the time of intervention by VCV in preventing maternal–fetal transmission following primary CMV infection in early pregnancy. A secondary aim was to evaluate the kinetics of viral load in response to treatment.

## METHODS

This retrospective, observational dual-center study included all pregnant patients referred for primary maternal CMV infection to 2 tertiary perinatal centers in the Paris area between November 2014 and September 2024. Some patients (ie, 34 patients) from this cohort were included in a prior study [5]. At the time of the study, routine universal CMV screening during pregnancy was not recommended in France but could be performed on an individual basis; screening uptake increased over time and varied regionally and was likely around 50% in the Paris area [6, 7].

All women referred to the participating perinatal centers for suspected primary CMV infection during the periconceptional period or first trimester of pregnancy were considered for inclusion. Women were included if primary CMV infection was subsequently confirmed and if maternal CMV viral load was available at the time of initial evaluation in the perinatal center.

The initial suspicion of primary CMV infection was based on serological screening (CMV IgG, IgM, and IgG avidity tests) performed by referring physicians. Confirmation and dating of maternal PI were subsequently assessed using serological results and standardized criteria, as previously described, and performed in a National Reference Laboratory [8]. Diagnosis of CMV PI during pregnancy relies on serology: detection of specific CMV-IgG and IgM, associated with CMV-IgG avidity in case of positive CMV-IgM [8–10]. For CMV-IgG avidity, specificity and sensitivity are comprised between 94% and 100% depending on the assay [11–16]. Cytomegalovirus serology (both IgG and IgM) was performed on serum collected in pregnant women during first trimester of pregnancy. Commercial immunoassays used were Architect, Alinity i (Abbott Diagnostics), VIDAS (bioMérieux, France), Elecsys (cobas e411, e601, e602, e402, e801) (Roche Diagnostics, Germany), Immulite 2000 (Siemens HealthCare, Germany), Access (Beckman-Coulter), and LXL (DiaSorin®, Saluggia, Italy). Cytomegalovirus IgG avidity was performed with both LXL (DiaSorin®, Saluggia, Italy) and VIDAS (bioMérieux, Craponne, France) in order to accurately date PI [16].

Cytomegalovirus PI < 1 month was defined by at least one of the following criteria:

- CMV-IgG avidity < 20% on the VIDAS assay and <0.1 on the LXL assay
- Evidence of recent IgG seroconversion (2 samples collected 1 months apart)

Cytomegalovirus PI < 2 months was defined by at least one of the following criteria:

- CMV-IgG avidity < 40% on the VIDAS assay and <0.3 on the LXL assay
- Documented CMV-IgG seroconversion on 2 samples collected 2 months apart

We deliberately restricted the analysis to certain recent CMV PI. In case of positive CMV-IgG/positive CMV-IgM/moderate CMV-IgG avidity (LXL < 0.4 and VIDAS > 0.40 but <0.65), a recent CMV PI (2–3 months) cannot be excluded. However, patients with this serological profile on the first available sample were excluded from the study because dating PI is often questionable in this situation.

Infections were classified as periconceptional if they occurred within 4 weeks before or after the estimated date of conception and as first-trimester infections if they occurred between 4 and 14 weeks of gestation (WG). Each case was reviewed and validated by the same virologist to ensure consistency in the confirmation and timing of PI.

Maternal CMV viral load was measured by quantitative PCR on whole blood at the time of initial evaluation in the perinatal center, prior to treatment initiation.

Patients were excluded if PI could not be confirmed, if infection occurred more than one month before conception, if they were referred due to ultrasound abnormalities suggestive of congenital CMV infection, or if the initial maternal viral load sample was collected more than 4 months after the estimated date of PI or after initiation of VCV treatment.

Valacyclovir treatment was offered to patients based on the benefit/risk information available at the time of consultation. From 2019 onwards, following the publication by Shahar-Nissan et al [2], VCV was systematically offered to pregnant women in cases of CMV PI. Previously, VCV was discussed on a case-by-case basis. Patients who received VCV constituted the treatment group and those who did not receive VCV constituted the untreated group. Treatment was administered orally at a high dose (8 g/day in 4 divided doses). Amniocentesis was offered to all patients, and when performed, it was conducted at least 6 weeks after the onset of infection and after 21 WG, in accordance with standard practice. Valacyclovir treatment was stopped if the amniotic fluid PCR CMV result was negative. For patients who did not undergo amniocentesis, treatment was systematically stopped at 21 WG. During VCV treatment, patients were monitored clinically and biologically (blood count, liver function, and renal function) every 2 weeks. For all patients with positive viral load, follow-up viral load monitoring was performed, regardless of whether they received treatment. Fetal ultrasounds were performed monthly and at birth. Congenital infection was assessed at birth by CMV PCR in urine collected in the first week of life and also in case with a positive amniocentesis.

The primary outcome was the diagnosis of congenital CMV infection at birth, assessed based on the initial viral load measured at the time of the patient's first evaluation. Secondary outcomes were kinetics of viral load during follow-up according to treatment status, as well as the risk of maternal–fetal transmission based on viral load kinetics in treated patients.

### CMV DNA Detection and Quantification

Cytomegalovirus DNA was detected and quantified in whole blood collected in EDTA tubes using quantitative real-time PCR (qPCR). DNA extraction was performed with the automated QIAsymphony RGQ system (Qiagen), and amplification was carried out using the artus CMV QS-RGQ kit on a Rotor-Gene Q thermocycler, according to the manufacturer's instructions. Appropriate positive, negative, and internal controls were included in each run. Maternal viral load was considered “negative” when the result was equal to 0 copies/mL, “detectable” but below the lower limit of quantification (LLOQ) when the result was between 1 and 200 copies/mL, and “quantifiable” when the result was >200 copies/mL (corresponding to 149 IU/mL or 2.17 log IU/mL). For statistical analyses, values below the lower limit of quantification were imputed as half the LLOQ.

### Statistical Analysis

Continuous variables were expressed as medians with interquartile ranges (25th–75th percentiles) and categorical variables as counts and percentages. Group comparisons were performed using Fisher's exact test because of the small sample sizes. The primary analysis included all participants and compared transmission rates between treated and untreated groups according to maternal viral load. A secondary analysis was conducted in subgroups defined by the timing of PI (periconceptional or first trimester).

Viral loads, expressed as log IU/mL, were compared between groups using the Mann–Whitney test to assess differences in median values and distribution. Viral kinetics were analyzed by simple linear regression of viral load over time within each group. The slope of the regression line represented the rate of change in viral load, and comparisons of slopes between groups were performed using analysis of variance to evaluate differences in viral dynamics.

All statistical analyses were performed using Stata software (StataCorp LLC, College Station, TX, USA) and GraphPad Prism (GraphPad Software, San Diego, CA, USA). A 2-sided *P* < .05 was considered statistically significant.

## RESULTS

**Figure 1.**
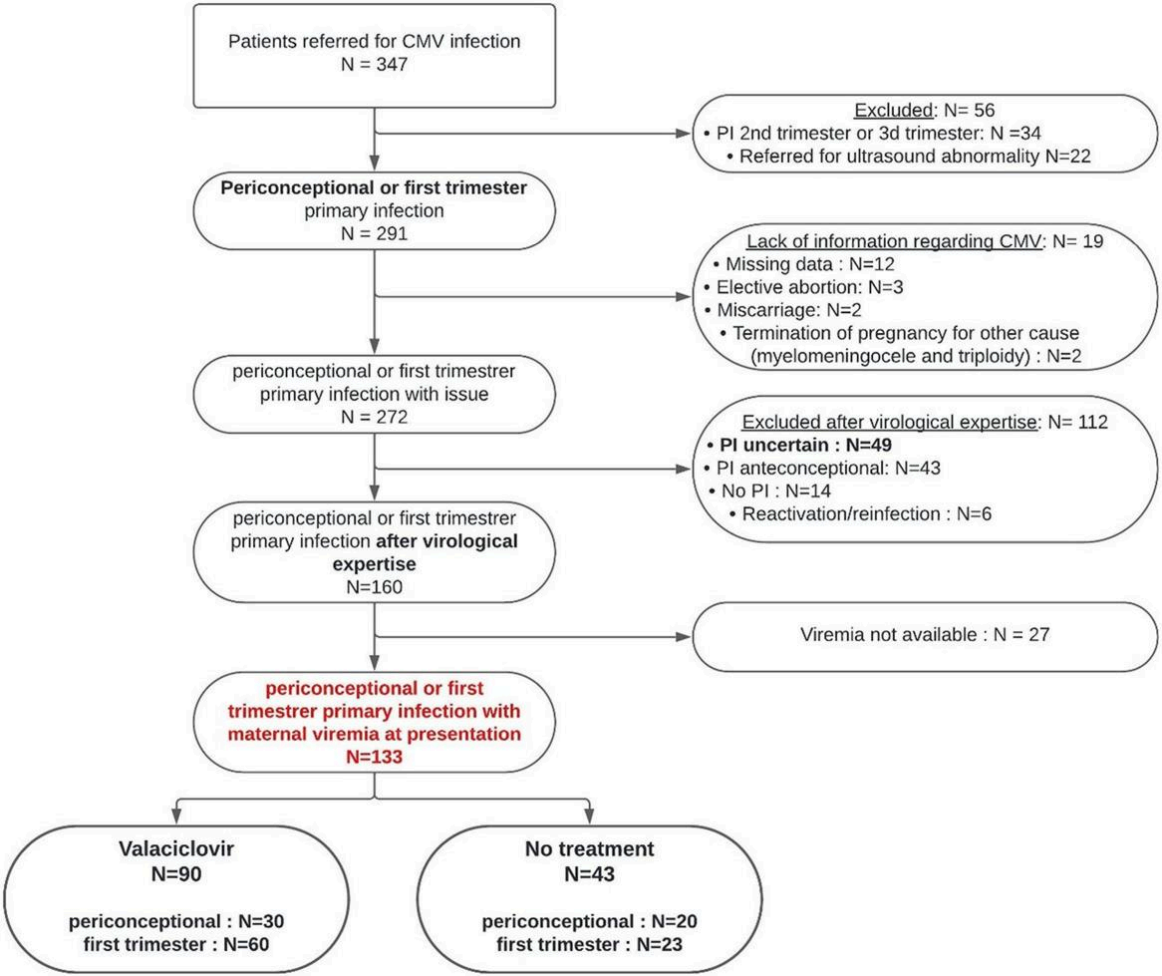
Flow chart of women with CMV primary infection (PI) included in the study.

Between 2014 and 2024, a total of 339 pregnant women were referred for CMV infection during pregnancy. Among them, 133 were finally included for the study (Figure 1), 90 patients received VCV (38% periconceptional infections and 62% first-trimester seroconversions), and 43 women did not receive VCV, of whom 46% periconceptional infections and 53% first-trimester seroconversions).

Maternal and obstetric characteristics are presented in Table 1. Patients in the VCV group were more likely to have detectable and quantifiable viral load at primary maternal infection diagnosis than those in the no treatment group (47% vs 27%).

**Table 1.** Maternal and Cytomegalovirus (CMV) Primary Infection Characteristics in Pregnancies

Characteristic	VCV	No Treatment	Total
	(n = 90)	(n = 43)	(n = 133)
Periconceptional primary infection, n (%)	30 (33%)	20 (46%)	50 (38%)
First-trimester primary infection, n (%)	60 (67%)	23 (53%)	83 (62%)
Median age (years)	32 (30–34)	32 (30–34)	32 (32–34)
Median gestational age (GA)			
At primary infection	4 (1–9)	5 (2–9)	5 (2–9)
At first viremia	13 (11–15)	15 (12–18)	14 (11–16)
Median interval between primary infection and viremia (weeks)	8 (5–11)	9 (7–12.5)	8.5 (5.5–12)
Median viral load if detectable or quantifiable (log IU/mL)	2.2 (1–3)	1.7 (1–2.6)	2.2 (1–2.8)
Median interval between viremia and initiation of VCV treatment (weeks)	0 (0–0.2)	NA	NA
Median interval between primary infection and initiation of VCV treatment (weeks)	7.5 (5–11.3)	NA	NA
Viral load status			
Negative	14 (15%)	17 (40%)	31 (24%)
Detectable	34 (38%)	14 (33%)	48 (36%)
Quantifiable	42 (47%)	12 (27%)	54 (40%)
Amniocentesis performed	85 (94%)	30 (70%)	115 (86%)
Periconceptional seroconversion	29 (34%)	15 (50%)	44 (38%)
First-trimester seroconversion	56 (66%)	15 (50%)	71 (62%)
Median gestational age at amniocentesis (WG)	21 (21–22)	22 (20–22)	21 (21–22.1)

Data are given as median (interquartile range) or n (%).

Abbreviations: GA, gestational age; VCV, valacyclovir.

**Figure 2.**
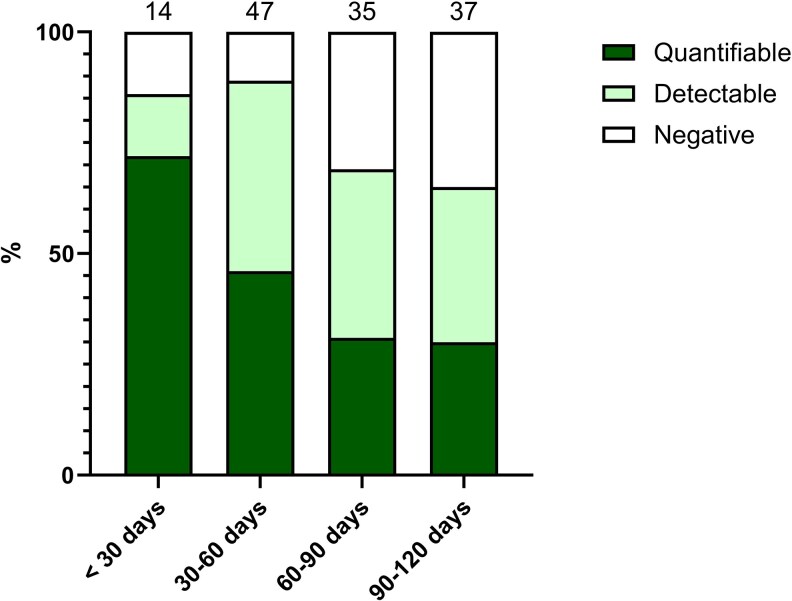
Maternal CMV viral load according to time between primary infection and first viral load. The number above each bar corresponds to the number of patients in each group.

When viral load was assessed within 30 days of seroconversion, 86% of patients (12/14) had detectable viral load (72% of which was quantifiable). Between 30- and 60-day post-seroconversion, 89% (42/47) had detectable viral load (which was quantifiable in 46%). From 60 to 90 days, 69% (24/35) had detectable viral load (which was quantifiable in 31%). Finally, between 90- and 120-day post-seroconversion, 65% (24/37) had detectable viral load (which was quantifiable in 30%) (Figure 2).

**Figure 3.**
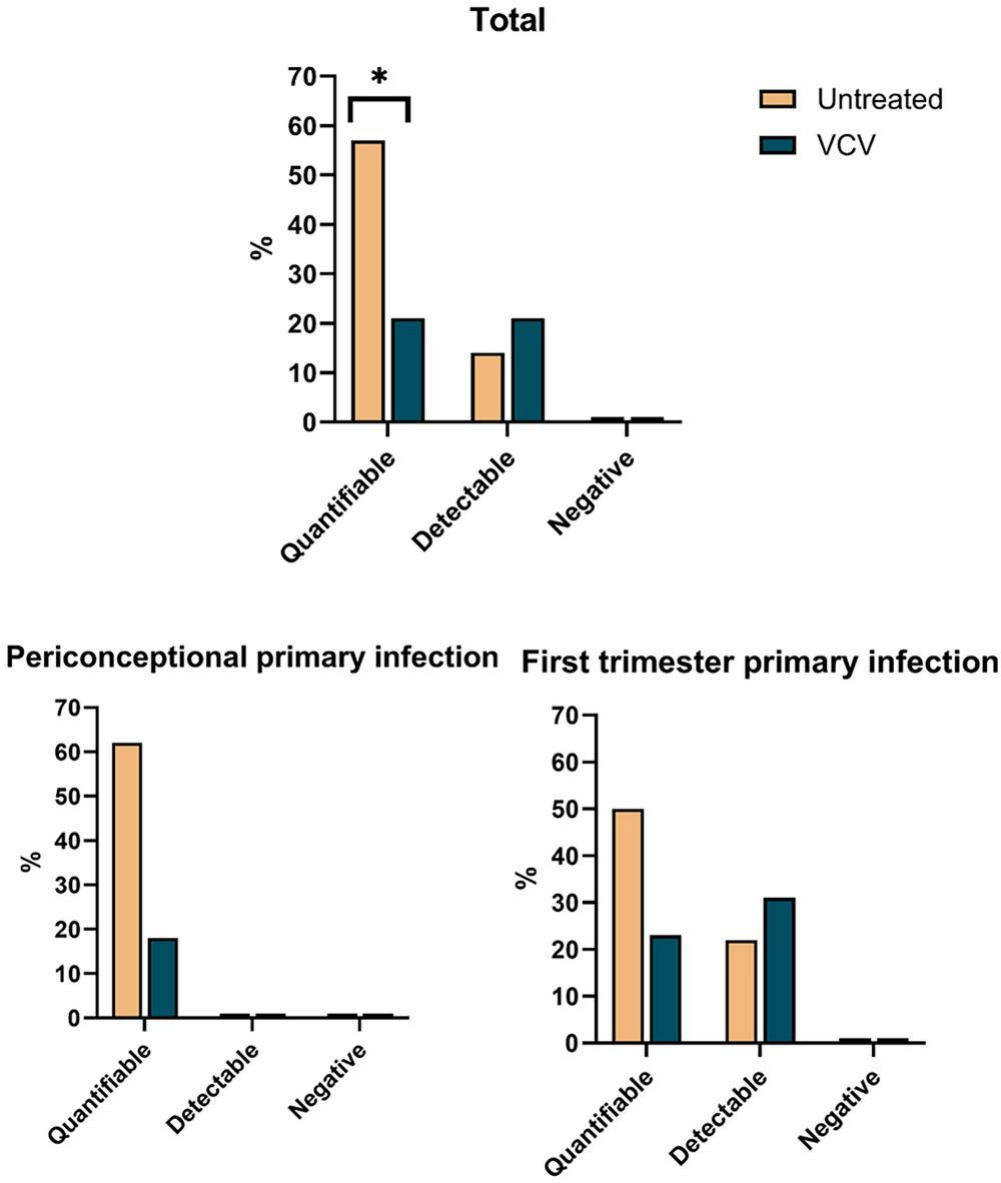
Maternal-to-fetal CMV transmission rate following maternal primary CMV infection, stratified by maternal viral load at referral. Untreated patients are shown in yellow, and valacyclovir-treated patients are shown in blue. An asterisk (*) indicates a statistically significant difference between groups (*P* < .05).

No case of maternal–fetal transmission was observed in the group with negative maternal viral load within 3 months of seroconversion, regardless of treatment with VCV (0/14 in the VCV group and 0/17 in the untreated group) (Figure 3). In cases where viral load was detectable, transmission rates demonstrated a minor, statistically nonsignificant difference between the groups: 21% (7/34) in the VCV group, compared to 14% (2/14) in the untreated group (*P* = 1.00). Finally, when viral load was quantifiable, maternal–fetal transmission was significantly reduced in the VCV-treated group compared to the untreated group (9/42, 21% vs 7/12, 58%; *P* = .028) (Figure 3).

When the subgroup with quantifiable viral load is analyzed according to the time of PI, the results seem similar (Figure 3). For periconceptional PI, when maternal viral load was quantifiable, the transmission rate was 18% (2/11) in the VCV group compared to 62% (5/8) in the untreated group (*P* = .07). For first-trimester seroconversion, when maternal viral load was quantifiable, the transmission rate was 23% in the VCV group (7/31) compared to 50% in the untreated group (2/4) (*P* = .268).

Among the patients with an initially positive viral load (102/133, 77%), 62% (63/102) underwent longitudinal monitoring of viral load kinetics over time. The initial viral load was, on average, slightly higher in the VCV group (2.3 vs 1.7 log UI/mL, *P* = .002). The follow-up viral load measurement used to assess viral load kinetics was performed on average 30 days after the initial viral load obtained at the first clinical evaluation.

**Figure 4.**
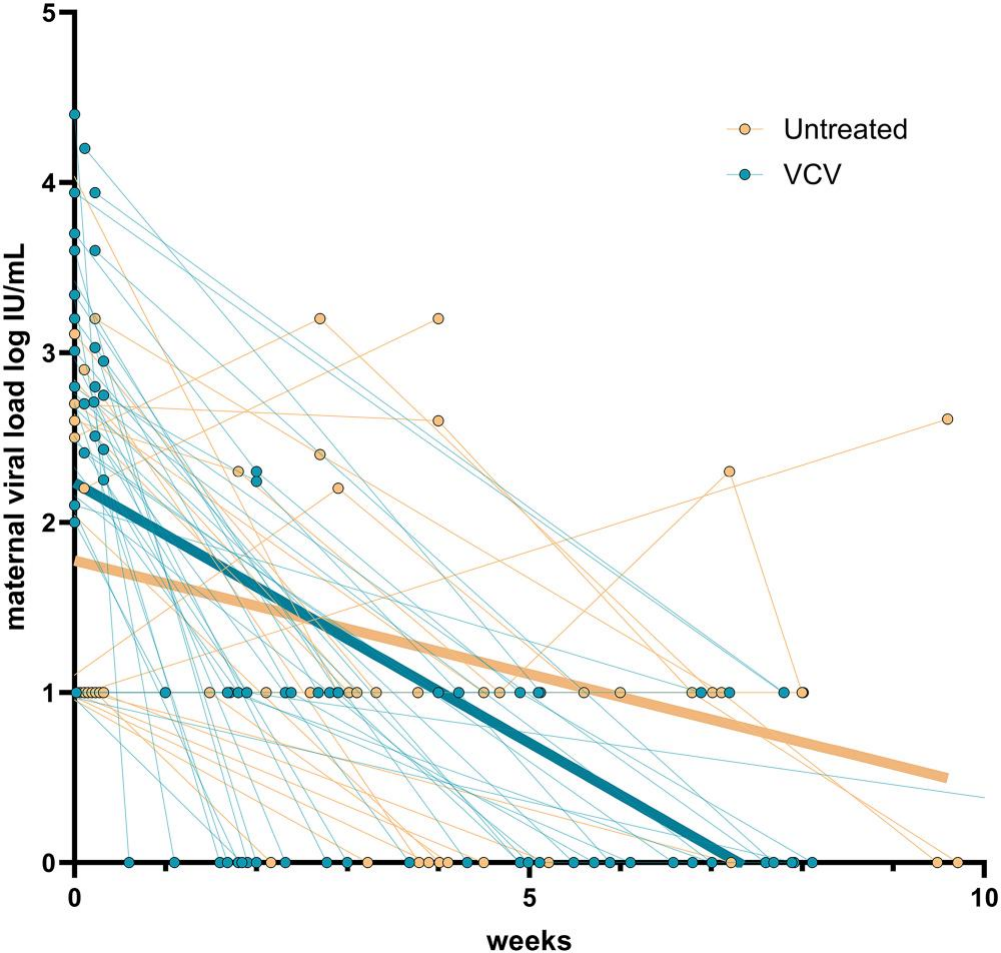
Kinetics of maternal CMV viral load during follow-up. The bold line represents the simple linear regression for each group (Y = −0.3059X + 2.233 for VCV group and Y = −0.1334X + 1.776 for untreated group).

The linear regression analysis of viral kinetics highlights a distinct difference between the treated and untreated groups. The decrease in viral load was significantly greater in the treated group compared to the untreated group (slope of −0.3059 in the VCV group vs −0.1334 in the untreated group, *P* = .003) (Figure 4).

## DISCUSSION

### Main Findings

Valacyclovir was associated with a reduced risk of maternal–fetal transmission following first-trimester or periconceptional PI, specifically in patients with quantifiable viral load. Furthermore, when viral load was undetectable at the time of initial evaluation, the risk of maternal–fetal transmission was very low. The reduction in maternal–fetal transmission was associated with a more rapid decline in maternal viral load in patients treated with VCV.

### Findings in View of Previous Studies

This study contributes to advancing the understanding of the prognostic significance of maternal viral load at the time of diagnosis (before treatment was initiated) on maternal–fetal transmission of CMV.

The prognostic value of maternal viral load after primary CMV infection has previously been investigated in untreated patients by Zavattoni et al, who demonstrated that viral load kinetics (clearance, persistence, or increase) were associated with the risk of maternal–fetal transmission [17]. Peak viremia is thought to occur within approximately 15 days after infection, followed by a gradual decline with inter-individual variability over several months [11, 18–20]. Our findings regarding the distribution of maternal CMV viremia over time are consistent with previously reported viral kinetics in pregnant women. Our findings extend this understanding by evaluating viral load dynamics in the context of high-dose VCV treatment. We observed a consistent reduction in maternal viral load under therapy, with 60% of women achieving a negative viral load within the first month of treatment, supporting a potential mechanism of action of VCV through suppression of maternal viremia.

Overall, the available literature suggests that detectable maternal viral load is associated with an increased risk of transmission; however, comparisons across studies should be interpreted with caution due to differences in sampling time and biological material. In several reports, viral load was measured at the time of amniocentesis or retrospectively using stored serum samples, which may not reflect early viral kinetics after PI [18, 21, 22]. In addition, the sensitivity of CMV PCR differs between serum and whole blood, particularly according to time from infection, with higher detection rates and viral loads generally observed in whole blood during early infection [18].

### Clinical Implications

The main prognostic factor found in previous studies for VCV efficacy in the secondary prevention of maternal–fetal transmission was the timing of treatment initiation [2]. The use of viral load appears to be relevant for the management of patients with CMV seroconversion. Based on our data regarding viral load kinetics in treated versus untreated patients, our main hypothesis is that VCV reduces maternal viral load, thereby shortening placental and fetal CMV exposure and consequently lowering the risk of transmission. This may suggest that maternal–fetal transmission could have already occurred in cases where VCV fails. For cases where viral load is detectable but unquantifiable, treatment initiation may occur too late to significantly reduce transmission risk.

Thus, we propose that rather than focusing on the trimester of PI (ie, periconceptional or first trimester), it is more important to consider maternal viral load early in pregnancy, particularly for periconceptional seroconversions, for which definitions vary across studies. In our study, when viral load was negative in whole blood within 3 months of seroconversion (on average 8.5 weeks after seroconversion), no case of maternal–fetal transmission occurred. Although it is challenging to extrapolate a complete absence of risk from negative viral load due to the limited number of patients, we hypothesize that some patients have transient viral load lasting only a few weeks, conferring a very low risk of transmission.

The relatively high transmission rate (ie, 58% vs 40% in the meta-analysis) observed in the untreated group with quantifiable viral load can be explained by the highly selected nature of this population: all women had rigorously confirmed and expert-reviewed PIs, and the requirement for detectable and quantifiable viral load identified a subgroup with an intrinsically higher risk of mother-to-child transmission [23].

Given that VCV acts by reducing maternal viral load to reduce the risk of mother-to-child transmission, its utility is questionable for secondary prevention in patients who present with negative viral load at the initiation of therapy. This consideration is particularly relevant in instances of periconceptional seroconversion, where a longer interval typically exists between maternal infection and the initiation of antiviral treatment. We therefore propose that CMV PCR testing on whole blood should be included in the diagnostic work-up for suspected maternal CMV PI during early pregnancy.

### Research Implications

Assessing maternal viral load could potentially contribute to the development of optimized screening and management strategies. Therefore, the inclusion of blood PCR in the panel of virological markers for diagnosing CMV PI should be considered. This could be challenging because the preferred sample for CMV PCR is whole blood, whereas serology is performed on serum. It would therefore require a new sample to be collected shortly after the diagnostic serology. It was previously considered to perform CMV PCR on serum to address this issue, but although it can be helpful, it is certainly less sensitive than PCR on whole blood [18].

Faas et al and Bourgon et al explored the feasibility of detecting CMV DNA in the first trimester of pregnancy during noninvasive fetal aneuploidy testing [24, 25]. While promising, the results are not yet clinically applicable and require further refinement.

Additionally, in cases of non-PI, first-trimester viral load assessment could be considered as a screening tool for identifying immune patients early in pregnancy who are at risk of maternal–fetal transmission. This approach warrants further investigation. In patients with prior immunity, serological testing is not useful for diagnosing secondary CMV infection [26]. In a retrospective case-control study involving women with preexisting CMV immunity at the onset of pregnancy, Huang et al demonstrated that the presence of CMV viral load was associated with an increased risk of congenital CMV (cCMV) infection, with odds ratios of 5.7, 6.5, and 13.0 for early, mid-, and late pregnancy, respectively [27].

### Strengths and Limitations

One of the main strengths of our study is that all maternal primary CMV infections were confirmed and dated by a virologist from a national expert center, ensuring the standardization and reliability of the PI dating.

The primary limitation of the study is its retrospective observational design. In the absence of a standardized management protocol, follow-up viral load assessments were not always performed at consistent intervals, and slight differences in baseline viral loads between groups may partly explain the faster decline in viral load observed among treated mothers, limiting the interpretation of viral load kinetics. However, the timing of assessments was similar between the 2 groups. Furthermore, quantitative PCR was performed, but patients were categorized into 3 groups (negative, detectable, and quantifiable) based on clinically relevant thresholds. This approach reflects real-life practice and allows for standardized interpretation.

Moreover, it appears that before the publication of Shahar-Nissan et al's pivotal randomized trial, some patients were treated with VCV on a case-by-case basis for the prevention of maternal–fetal transmission. The decision to initiate treatment may have been influenced by viral load. Notably, patients with positive maternal viral load were more frequently treated with VCV. Consequently, a higher proportion of patients with positive maternal viral load were observed in the treated group, which explains why patients in the VCV group were more likely to have quantifiable viral load than those in the untreated group (47% vs 27%).

Finally, multivariable analysis was not performed, as in our cohort, only maternal age at the time of infection could be considered a relevant confounder. Including a limited number of covariates would have markedly reduced statistical power. We therefore opted for subgroup analyses which, although not statistically significant, consistently demonstrated similar trends, supporting the validity of our findings.

## CONCLUSION

Maternal viral load emerged as a valuable prognostic marker because (1) it allows for the prediction of a very low risk of maternal–fetal transmission when viral load is negative and (2) it supports the potential benefit of VCV treatment for secondary prevention of maternal–fetal transmission, particularly when viral load is quantifiable.
